# The diagnostic value of CT-guided percutaneous lung biopsy in atypical pulmonary hamartoma and pulmonary sclerosing pneumocytoma

**DOI:** 10.3389/fonc.2025.1653012

**Published:** 2025-12-15

**Authors:** Meng Li, Yang Li, Xiaopeng Zhao, Xiangming Wang, Gaofeng Shi, Andu Zhang, Huiyan Deng, Jialiang Ren

**Affiliations:** 1 Department of Computed Tomography and Magnetic Resonance Imaging, The Fourth Hospital of Hebei Medical University, Shijiazhuang, Hebei, China; 2 Department of Thoracic Surgery, The Fourth Hospital of Hebei Medical University, Shijiazhuang, Shijiazhuang, Hebei, China; 3 Department of Radiotherapy, The Fourth Hospital of Hebei Medical University, Shijiazhuang, Hebei, China; 4 Department of Pathology, The Fourth Hospital of Hebei Medical University, Shijiazhuang, Hebei, China; 5 Department of Pharmaceuticals Diagnostics, GE Healthcare China, Beijing, China

**Keywords:** CT-guided, percutaneous lung biopsy, atypical pulmonary hamartoma, pulmonary sclerosing pneumocytoma, nodule

## Abstract

**Objective:**

This study aims to evaluate the clinical utility of computed tomography-guided percutaneous lung biopsy (CT-PLB) in the diagnosis of atypical pulmonary hamartoma (APH) and pulmonary sclerosing pneumocytoma (PSP).

**Methods:**

This retrospective study analyzed 19 patients with pulmonary nodules who underwent CT-PLB at our hospital between October 2016 and August 2019. All patients underwent surgical excision within two weeks following CT-PLB, and the postoperative histopathological results were used as the reference standard for diagnosis. Among these patients, ten cases were confirmed as PSP and assigned to the PSP group, while nine cases were diagnosed as pulmonary hamartoma and assigned to the APH group. The diagnostic accuracy of CT-PLB for APH and PSP, as well as the incidence of procedure-related complications, were analyzed and compared.

**Results:**

Among the 19 patients, the overall diagnostic accuracy of CT-PLB was 89.5% (17/19). The diagnostic accuracy was 88.9% (8/9) in the APH group and 90.0% (9/10) in the PSP group. Complications included one case of minimal pneumothorax in the PSP group and one case of mild hemoptysis in the APH group. Additionally, 14 patients experienced mild to moderate pulmonary hemorrhage, including 8 cases (5 mild, 3 moderate) in the APH group and 6 cases (all mild) in the PSP group. No severe complications, such as pleural reactions, occurred in any patient.

**Conclusion:**

CT-PLB demonstrates high clinical diagnostic value for both APH and PSP. The procedure is safe, reliable, and associated with few complications. Thus, it can serve as a valuable tool for preoperative clinical diagnosis.

## 1 Introduction

Pulmonary hamartoma (PH) is a benign tumor originating from mesenchymal tissue and represents the most common benign lung tumor, accounting for approximately 70% of all benign pulmonary neoplasms (1). PH is characterized by a heterogeneous assemblage of tissues, typically encompassing cartilage, fat tissue, fibrous tissue, and smooth muscle, resulting in diverse computed tomography (CT) manifestations (2). Characteristic CT features of PH include the presence of intranodular fat and “popcorn-like” calcifications (3). The variable proportions of fat and/or cartilage within PH can lead to diverse attenuation patterns on CT, resulting in atypical CT characteristics (4). Consequently, such lesions can be classified as atypical pulmonary hamartomas (APH) (5), posing challenges for clinical diagnosis and differential diagnosis. Pulmonary sclerosing pneumocytoma (PSP), previously referred to as pulmonary sclerosing hemangioma, is a rare benign lung tumor that predominantly affects Asian women (6). It accounts for approximately 10.7% of benign pulmonary neoplasms, ranking second only to PH (7, 8). On CT images, PSP typically presents as a well-defined solitary nodule with relatively homogeneous density. The lesion may exhibit punctate calcifications and marked contrast enhancement, often characterized by a prolonged enhancement. Common CT features include peripheral vascular tail signs and halo signs (9, 10). However, these characteristics are not pathognomonic and may be absent or less conspicuous in certain cases (10, 11).

PH is generally a benign neoplasm characterized by slow growth. Consequently, a conservative management strategy involving regular imaging follow-up is typically employed, with surgical resection reserved for cases presenting clinical symptoms or significant of the lesion (12, 13). In contrast, although pulmonary sclerosing pneumocytoma (PSP) is also classified as a benign tumor, it possesses low-grade malignant potential and may cause local compressive symptoms. Therefore, active surgical intervention aimed at complete resection is often preferred to reduce the risk of recurrence (14, 15). Given these distinct clinical characteristics, accurate differentiation between PH and PSP is crucial for guiding appropriate treatment strategies. CT-guided percutaneous lung biopsy (CT-PLB) is a minimally invasive, precisely localized diagnostic procedure that enables acquisition of representative tumor tissue under imaging guidance, thereby providing reliable pathological confirmation (16, 17). However, due to the rarity of both neoplasms, there have been no studies evaluating the utility of CT-PLB in the diagnosis and differential diagnosis of APH and PSP.

This study aims to evaluate the utility of CT-PLB in the diagnosis of APH and PSP, thereby providing clinicians with a safe and reliable diagnostic tool to personalized treatment strategies guided by accurate pathological diagnoses.

## 2 Materials and methods

### 2.1 Study population

This retrospective study was approved by the Ethics Committee of our hospital, and the requirement for written informed consent was waived. Patients who underwent contrast-enhanced computed tomography (CECT) and CT-PLB for pulmonary nodules at our hospital between October 2016 and August 2019 were retrospectively identified. All enrolled patients subsequently underwent surgical resection, with postoperative pathological examination confirming a diagnosis of PH or PSP.

The inclusion criteria were as follows: (1) pathological diagnosis of PH or PSP confirmed by postoperative histopathological examination; (2) presence of atypical imaging features on preoperative CECT images, leading to diagnostic uncertainty; and (3) successful acquisition of adequate pathological specimens from pulmonary nodules via CT-PLB prior to surgery. The exclusion criteria were as follows: (1) absence of available CECT images in the Picture Archiving and Communication System (PACS) or incomplete imaging data precluding comprehensive analysis; and (2) incomplete clinical or pathological records, rendering patient data insufficient for analysis. Among 3, 158 patients who underwent CT-PLB, 19 patients were enrolled following a rigorous screening process. Each enrolled patient had a solitary lesion, resulting in the final evaluation of 19 lesions.

### 2.2 Preoperative preparation for CT-PLB

All patients underwent CECT prior to biopsy to accurately assess tumor size, number, and location, thereby confirming the target tumor. A personalized CT-PLB plan was subsequently developed based on each patient’s individual condition, including optimal positioning, selection of needle entry site, and identification of potential complications, with corresponding preparations made accordingly. A comprehensive pre-biopsy evaluation was conducted, including assessments of liver and renal function, electrocardiogram (ECG), complete blood count (CBC), and coagulation profile, to determine patients’ overall health status and eligibility for the procedure. Detailed explanations of the biopsy process, associated precautions, potential complications, and management strategies were provided to ensure that patients and their families fully understood the procedure. Informed consent was subsequently obtained.

Additionally, patients were instructed in breathing exercises designed to minimize movement during the biopsy. Specifically, they practiced breath-holding for 10 to 15 seconds while relaxed, followed by slow and even breathing, thereby reducing the risk of coughing or sudden movements during the procedure.

### 2.3 CT-PLB procedure

All biopsies were performed by a team of three interventional radiologists, each with over four years of experience in CT-PLB. The procedures were carried out using a 16-slice spiral CT scanner (SOMATOM Emotion 16, Siemens Healthcare, Erlangen, Germany). Pre-biopsy thin-slice CECT images were utilized to determine the optimal patient positioning—supine, prone, or lateral—based on the nodule’s location and the patient’s overall condition. Patients were then positioned accordingly on the CT examination table, and axial chest CT scans were acquired with a slice thickness of 3 mm and a reconstruction thickness of 1.5 mm.Three-dimensional (3D) reconstructions were performed to evaluate the lesion’s location, size, spatial relationship with adjacent vessels, and distance from the chest wall. Additionally, the depth from the edge of the nodule to the skin surface was measured.

The procedure was meticulously designed to circumvent bony structures, blood vessels, pulmonary bullae, and fissures, prioritizing the shortest and safest trajectory based on the nodule’s largest cross-sectional area. Consequently, the puncture trajectory, angle, and depth were systematically determined. The puncture site was accurately identified using laser positioning lines and surface locating grids, and subsequently marked with a skin marker. Following comprehensive disinfection and the application of a sterile surgical drape, local anesthesia was administered using lidocaine. The needle used for the administration of local anesthetic lidocaine was retained at the subcutaneous puncture site to serve as a marker, facilitating precise alignment of the puncture trajectory and angle. This practice effectively minimizes the CT scanning range, thereby reducing the patient’s exposure to radiation.

Subsequently, the coaxial biopsy needle was advanced into the lesion, and one to four tissue samples were obtained using the BioPince™ 18-gauge full core biopsy instrument (BioPince™, Argon Medical Devices, Inc., USA). The specimens were immediately fixed in 10% formalin for pathological examination. If the sample was insufficient, the puncture path could be adjusted for repeat sampling.

Throughout the procedure, the patient’s vital signs were closely monitored. Patients were instructed to hold their breath during needle withdrawal to reduce the risk of complications. Following the biopsy, a chest CT scan was performed to assess potential complications, including pneumothorax and hemorrhage. The puncture site was compressed for 1 to 2 minutes and then bandaged. Patients were observed for 4 hours post-biopsy, during which vital signs and oxygen saturation were continuously monitored. They were advised to avoid strenuous activities and coughing after the biopsy. Minor pneumothorax typically does not require intervention; however, patients should limit physical activity for 24 hours to prevent exacerbation of the pneumothorax.

### 2.4 Data collection and statistical analysis

**Figure 1:**
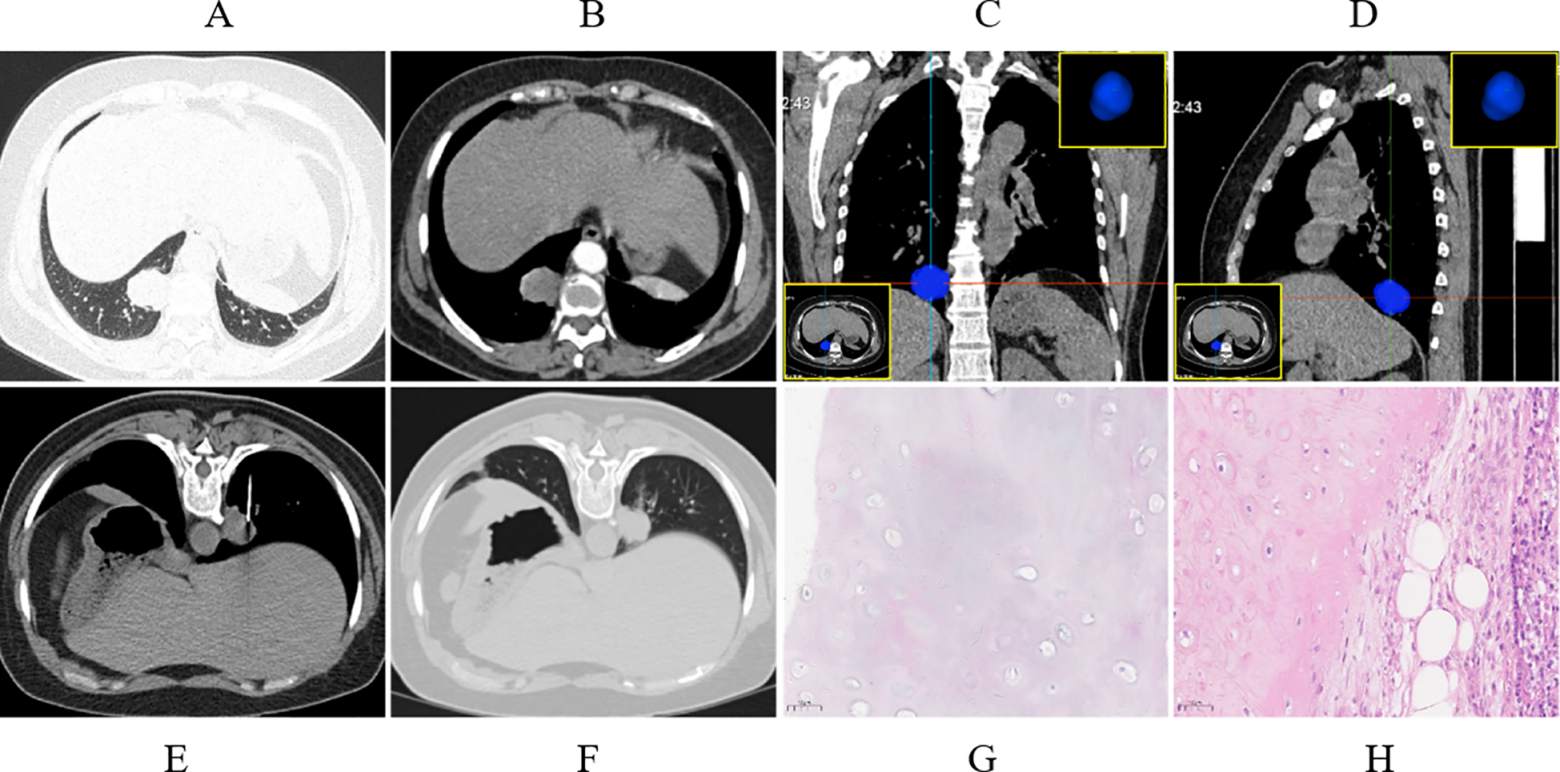
Case presentation. A 42-year-old female, presented with solid nodules in the right upper lobe. **(A)** Axial CT images showing a soft tissue nodule located in the posterior basal segment of the right lower lobe, measuring approximately 2.91 cm in the longest diameter (lung window). **(B)** Mild enhancement of the lesion observed during the arterial phase (mediastinal window). **(C, D)** Coronal and sagittal CT images were utilized to precisely delineate the location of the lesions. **(E)** The patient underwent CT-guided percutaneous biopsy in the prone position via a dorsal approach (mediastinal window). **(F)** Follow-up chest CT demonstrated minimal hemorrhage in the lung parenchyma surrounding the needle tract (lung window). **(G)** CT-guided biopsy pathology suggested a diagnosis of pulmonary hamartoma (PH) (Hematoxylin and Eosin [HE], 20× magnification). **(H)** Post-surgical resection pathology confirmed the diagnosis of pulmonary hamartoma (HE, 20× magnification).

**Figure 2:**
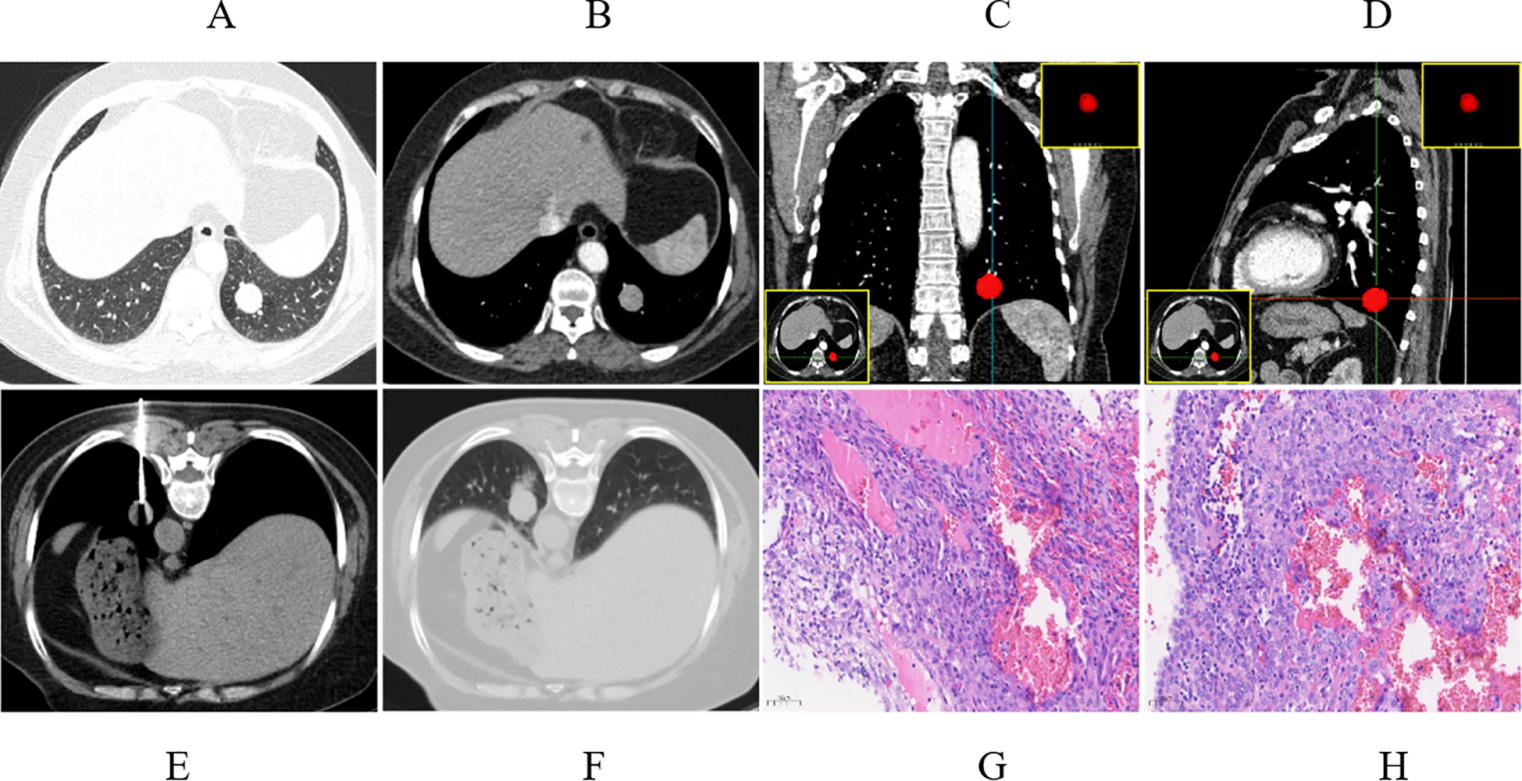
A 56-year-old female, presented with solid nodules in the left upper lobe. **(A)** Axial CT images of a soft tissue nodule in the posterior basal segment of the lower lobe of the left lung, measuring approximately 2.12 cm in length (lung window). **(B)** Significant enhancement of the lesion in the arterial phase (mediastinal window). **(C, D)** Coronal and sagittal CT images were utilized to precisely delineate the location of the lesions. **(E)** The patient underwent puncture biopsy in a prone position with dorsal entry (mediastinal window). **(F)** Follow-up chest CT revealed minimal bleeding in the lung tissue around the needle tract (lung window). **(G)** CT-guided pathological results of the puncture suggest the diagnosis of PSP (HE, 20×). **(H)** The pathological results post-surgical resection confirmed the diagnosis of PSP (HE, 20×).

The anatomical characteristics of the lesions were systematically documented utilizing preoperative CECT images. These characteristics encompassed the lesion’s location, the long and short diameters, the mean CT value on pre-contrast and arterial-phase images, the tumor morphology (e.g., lobulated or spiculated margins), and associated imaging signs such as vascular encasement. Additionally, parameters pertinent to the biopsy procedure were meticulously recorded, including the number of needle insertions, the quantity of biopsy samples obtained, the puncture position, and the types of complications encountered, such as pneumothorax, pulmonary hemorrhage, hemoptysis, and pleural reactions. The postoperative pathological results were considered the gold standard. The diagnostic accuracy of the biopsy pathology was ascertained by comparing the biopsy findings with the corresponding surgical pathology results, as illustrated in **Figures 1** and **2**.

Continuous variables that followed a normal distribution were reported as mean ± standard deviation (SD), and comparisons between groups were performed using independent samples t-test. Variables that did not adhere to a normal distribution were presented as median [interquartile range (IQR)] and assessed using the Mann-Whitney U test. Categorical variables were evaluated using either the chi-square test or Fishe’s exact test, depending on suitability. Comparative analyses of imaging features, biopsy accuracy, and complication rates between the APH group and the PSP group were performed. Statistical significance was established at P < 0.05. All statistical analyses were executed using R software (version 4.3.1).

## 3 Results

### 3.1 Clinical and CT characteristics

A total of 19 patients were enrolled in this study, with a mean age of 53.16 ± 8.52 years (range, 37–67 years), comprising 2 males and 17 females (**Table 1**). The APH group comprised 9 patients (2 males and 7 females) with a mean age of 52.22 ± 11.09 years, exhibiting an age range comparable to that of the entire study population. The PSP group consisted of 10 female patients, with a mean age of 54.00 ± 4.73 years (range, 48–64 years).

**Table 1:** Analysis of clinical and CT characteristics.

Clinical and CT characteristics	Groups			P-value
	Total(n = 19)	APH (n = 9)	PSP (n = 10)	
Age (years), mean (SD)	53.16 ± 8.42	52.22 ± 11.09	54.00 ± 4.73	0.683^@^
Gender, n (%)				0.211^#^
Male	2(10.5)	2(22.2)	0(0)	
Female	17(89.5)	7(77.8)	10(100.0)	
Zone location, n (%)				0.034^#^
Middle zone	5 (26.3)	1 (11.1)	4 (40.0)	
Inner zone	3 (15.8)	0 (0)	3 (30.0)	
Outer zone	11 (57.9)	8 (88.9)	3 (30.0)	
Lobe location, n (%)				0.163^#^
Right upper lobe	3 (15.8)	2 (22.2)	1 (10.0)	
Right middle lobe	3 (15.8)	1 (11.1)	2 (20.0)	
Right lower lobe	7 (36.8)	4 (44.4)	3 (30.0)	
Left upper lobe	2 (10.5)	2 (22.2)	0 (0)	
Left lower lobe	4 (21.1)	0 (0)	4 (40.0)	
Pre-contrast mean CT value (Hu), mean (SD)	27.70 ± 9.94	22.03 ± 7.37	32.80 ± 9.17	0.017^@^
Arterial phase mean CT value (Hu), median [IQR]	45.47[32.19, 61.24]	32.190[26.21, 37.25]	58.47[46.73, 83.53]	<0.001^&^
Long diameter (cm), median [IQR]	21.28[12.58, 28.32]	12.58[10.58, 22.75]	22.12[20.73, 39.45]	0.030^&^
Short diameter (cm), median [IQR]	18.94[9.79, 32.86]	9.79[9.41, 17.16]	21.13[18.94, 35.81]	0.016^&^
Morphology, n (%)				0.353^#^
Oval	3 (15.8)	1 (11.1)	2 (20.0)	
Rounded	12 (63.2)	6 (66.7)	6 (60.0)	
Irregular	2 (10.5)	0 (0)	2 (20.0)	
Lobulated	2 (10.5)	2 (22.2)	0 (0)	
Air crescent sign, n (%)				1.000^#^
Absent	18 (94.7)	9 (100.0)	9 (90.0)	
Present	1 (5.3)	0 (0)	1 (10.0)	
Vascular interface sign, n (%)				0.040^#^
Absent	8 (42.1)	6 (66.7)	2 (20.0)	
Present	11 (57.9)	3 (33.3)	8 (80.0)	

^@^independent samples t-test; ^&^Mannwhitney-U test; ^#^chi-square test or Fisher’s exact test.

In the APH group, the lesion distribution by lung lobe was as follows: right upper lobe (2 cases), right middle lobe (1 case), right lower lobe (4 cases), left upper lobe (2 cases), and left lower lobe (0 case). In terms of zone distribution, there were 0 cases in the inner zone, 1 case in the middle zone, and 8 cases in the outer zone. In the PSP group, lesions were located in the right upper lobe (1 case), right middle lobe (2 cases), right lower lobe (3 cases), left upper lobe (0 case), and left lower lobe (4 cases). Additionally, there were 3 cases in the inner zone, 3 cases in the middle zone, and 4 cases in the outer zone.

The quantitative CT analysis demonstrated that the mean CT value of the lesions on pre-contrast images was significantly lower in the APH group (22.03 ± 7.37 Hounsfield units [HU]) compared to the PSP group (32.80 ± 9.17 HU; P = 0.017). Similarly, the mean CT value of the lesions on arterial-phase differed significantly between the two groups (P < 0.001). The median long diameter of lesions was 12.58 cm (interquartile range [IQR], 10.58–22.75 cm) in the APH group, significantly smaller than that in the PSP group, which measured 22.12 cm (IQR, 20.73–39.45 cm; P = 0.030). The median short diameter was also smaller in the APH group at 9.79 cm (IQR, 9.41–17.16 cm) versus 21.13 cm (IQR, 18.94–35.81 cm) in the PSP group (P = 0.016).

“Vascular encasement” was observed in 3 of 9 cases (33.3%) in the APH group and 8 of 10 cases (80%) in the PSP group, representing a statistically significant difference (P = 0.040). Regarding lesion morphology, the APH group comprised 0 irregular, 1 oval, 6 round, and 2 slightly lobulated nodules, whereas the PSP group included 2 irregular, 2 oval, 6 round, and no slightly lobulated nodules. No statistically significant difference was foundin lesion morphology distribution between the two groups (P = 0.353).

Regarding comorbidities, the PSP group included one case each of breast cancer, gastrointestinal stromal tumor, and pulmonary adenocarcinoma, whereas the APH group had one case of breast cancer and two cases of pulmonary adenocarcinoma.

### 3.2 CT-PLB analysis and complications

As summarized in **Table 2**, in the APH group, 5 patients underwent the procedure in the supine position, 3 in the prone position, and 1 in the left lateral decubitus position. In the PSP group, 4 patients underwent the procedure in the supine position and 6 in the prone position. There was no statistically significant difference in patient puncture positioning and needle adjustment between the two groups. The median number of biopsy tissue samples obtained was 2.00 (interquartile range [IQR], 2.00–2.00) in the APH group and 2.00 (IQR, 2.00–3.00) in the PSP group, with no significant difference (P = 0.210).

**Table 2:** Analysis of CT-guided puncture and complications data.

CT-PLB and complications data	Groups			P-value
	Total(n = 19)	APH (n = 9)	PSP (n = 10)	
Position, n (%)				0.484^#^
Supine	9 (47.4)	5 (55.6)	4 (40.0)	
Prone	9 (47.4)	3 (33.3)	6 (60.0)	
Left Lateral	1 (5.2)	1 (11.1)	0 (0)	
Needle adjustment (n), mean (SD)	3.26 (1.52)	3.111 (1.29)	3.40 (1.69)	0.698^@^
Tissue sample (n), median [IQR]	2.00 (2.00, 3.00)	2.00 (2.00, 2.00)	2.000 (2.00, 3.00)	0.210^&^
Pulmonary Hemorrhage, n (%)				0.112^#^
None	5 (26.3)	1 (11.1)	4 (40.0)	
Minimal	11 (57.9)	5 (55.6)	6 (60.0)	
Moderate	3 (15.8)	3 (33.3)	0 (0)	
Hemoptysis, n (%)				0.474^#^
None	18 (94.7)	8 (88.9)	10 (100.0)	
Present	1 (5.3)	1 (11.1)	0 (0)	
Pneumothorax, n (%)				1.000^#^
None	18 (94.7)	9 (100.0)	9 (90.0)	
Present	1 (5.3)	0 (0)	1 (10.0)	

^#^chi-square test or Fisher’s exact test; ^@^independent samples t-test; ^&^Mann-Whitney U test.

Regarding complications, pulmonary hemorrhage occurred in both groups. In the APH group, there were 5 cases of minor bleeding and 3 cases of moderate bleeding, whereas in the PSP group, 6 cases were classified as minor bleeding. The difference in hemorrhage severity between the two groups was not statistically significant. Notably, no cases of pneumothorax were observed in the PSP group, while the APH group had 1 case of minor pneumothorax. Hemoptysis was reported in only 1 case in the APH group and was absent in the PSP group. Additionally, no pleural reactions were observed in either group.

### 3.3 CT-PLB pathology results and surgical pathology results

Among the 19 patients with pulmonary nodules, the puncture success rate was 100%. Using pathological findings from surgical resection as the gold standard, the overall diagnostic accuracy of CT-PLB was 89.5% (17/19). Specifically, the diagnostic accuracy was 88.9% (8/9) in the APH group and 90.0% (9/10) in the PSP group, with no statistically significant difference observed between the two groups.

In the APH group, one false-negative case was recorded, where the biopsy specimen contained only a limited amount of lung tissue. Similarly, the PSP group had one false-negative case, in which the biopsy revealed atypical cells insufficient to establish a definitive diagnosis.

## 4 Discussion

PH and pulmonary PSP are the two most common benign lung tumors (8, 18). From a pathological perspective, the two tumors exhibit markedly different histological features. PH is characterized by a heterogeneous mixture of tissue components, including cartilage, adipose tissue, and fibrous tissue, that collectively form a distinctive “puzzle-like” architecture. In contrast, PSP displays highly variable histological morphology, predominantly comprising four distinct pathological patterns: papillary, solid, sclerotic, and hemorrhagic (19). Epidemiologically, PH is more commonly observed in middle-aged and elderly men (18), whereas PSP predominantly affects middle-aged women (19). On CT images, both PH and PSP typically manifest as well-defined solitary pulmonary nodules or masses without obvious lobulation or spiculation, and occasionally contain calcific foci (20). Notably, the presence of “popcorn” calcification on CT scans is considered a characteristic feature of PH (3, 21). Accurate identification of PH on imaging is crucial because it informs appropriate follow-up intervals and facilitates regular monitoring. However, these characteristic imaging features are observed in fewer than 30% of cases (3, 22), and and atypical presentations of pulmonary hamartomas can complicate diagnosis. Therefore, relying solely on morphological imaging characteristics is often insufficient to reliably differentiate PH from PSP.

There are significant differences in treatment strategies between PH and PSP. PH is typically a benign lesion characterized by slow growth, and its management primarily involves observation and regular follow-up; surgical resection is considered only when severe symptoms develop or if the lesion exhibits rapid growth (23). In contrast, although PSP is also benign, it has the potential for low-grade malignant behavior that may lead to local compressive symptoms. Therefore, early surgical intervention is often recommended to achieve complete resection and minimize the risk of recurrence (24–29). Accurate differentiation between the two lesions is crucial for formulating appropriate treatment plans, and robust diagnostic capabilities can effectively guide clinical decision-making. Our study revealed that in instances where imaging diagnostics fail to effectively distinguish between APH and PSP, the CT-PLB can serve as an potential approach for achieving a accurate diagnosis. This accurate diagnostic approach facilitates the development of targeted treatment strategies, thereby potentially delivering substantial clinical benefits to patients. Specifically, when CT-PLB confirms a diagnosis of APH and the lesion is small and asymptomatic, it is advisable to adopt a watchful waiting approach in order to avoid unnecessary surgical trauma. Conversely, for patients diagnosed with PSP, even in the absence of clinical symptoms, selective surgical intervention is recommended to mitigate the risk of future metastasis, considering the tumor’s malignant potential.

In recent years, with continuous advancements in medical technology, CT-PLB has been widely adopted for the qualitative diagnosis of pulmonary diseases, particularly in tumor diagnosis and differential diagnosis (30–34). CT-PLB is characterized by minimal lung tissue trauma, precise lesion localization, high safety, and a low incidence of complications (32, 35). CT imaging not only provides clear visualization of lesion location, size, and spatial relationship with surrounding tissues but also facilitates evaluation of necrotic areas within the lesion, thereby providing significant diagnostic advantages (16, 17, 35). The diagnostic accuracy of CT-PLB for pulmonary lesions is reported to range from 93% to 95% (36). Several studies have demonstrated that value of CT-PLB in the qualitative diagnosis of PH and PSP; however, comparative analyses regarding their diagnostic accuracy, complication rates, and distinguishing features remain limited (23, 37).

Our research findings demonstrate that among 19 cases of CT-PLB, the puncture success rate was 100%. Of these, 17 cases yielded biopsy results consistent with postoperative pathological results, resulting in an accuracy rate of 89.5% (17/19). In the APH group, one case resulted a false-negative outcome, where the biopsy specimen contained only a small amount of lung tissue. Retrospective analysis revealed that the tumor measured 1.04 cm and 0.93 cm in its long and short diameters, respectively, suggesting that the small tumor size may have contributed to inadequate lesion sampling. In a previous study, Tipaldi et al. (34) revealed that nodule size is a key imaging predictor of the diagnostic success rate of CT-PLB, with nodules smaller than 18 mm in diameter potentially yielding inconclusive histopathological results. Furthermore, the increased number of punctures necessitated by the small tumor size led to moderate pulmonary hemorrhage in this patient. In the PSP group, there was also one false-negative case, in which the biopsy revealed only a small number of atypical cells. Retrospective analysis revealed that the tumor measured 2.62 cm and 2.37 cm in its long and short diameters, respectively. The false-negative result may be attributed to the pronounced heterogeneity of the tumor, which caused the puncture site to miss characteristic PSP structures, thereby impeding accurate qualitative diagnosis.

Pneumothorax is generally recognized as the most common complication of CT-PLB, with reported incidence rates ranging from 17% to 40.4% (38–40). In our study, which included 19 patients, only a single instance of minimal pneumothorax was documented, occurring in the PSP group where the lesion was situated subpleurally. The notably low incidence of pneumothorax in our cohort may be attributed to the solid nature of both tumor types, which likely results in minimal disruption of lung parenchyma and bronchial structures, thereby reducing the risk of pneumothorax.

Pulmonary hemorrhage is another frequent complication of CT-PLB, with reported incidences ranging from 31.38% to 69% (41, 42). In our study, the incidence of pulmonary hemorrhage was 73.7% (14/19), including 11 cases of minimal hemorrhage and 3 cases of moderate hemorrhage. Notably, all three moderate hemorrhage cases occurred in the APH group. This may be attributed to the small lesion sizes, which necessitated a higher number of needle passes and sampling of larger lung tissue volumes with the biopsy gun. Among the three cases of moderate hemorrhage in the APH group, one patient exhibited hemoptysis. Retrospective analysis revealed that this nodule was located in the left upper lobe, with dimensions of 2.54 cm and 2.07 cm in its long and short diameters, respectively. The hemoptysis may resulted from interference from the rib overlying the lesion, which required multiple needle adjustment during the procedure. Additionally, three biopsy cutting procedures were performed to ensure adequate tissue sampling, resulting in greater lung tissue injury. Fortunately, the hemoptysis was mild and resolved spontaneously. The biopsy pathology suggested hamartoma, consistent with the final surgical pathology. In contrast, there were no patients with moderate hemorrhage in the PSP group, and no cases of hemoptysis were observed.

This study has several limitations. First, as a retrospective study, some follow-up CT and clinical data were incomplete, leading to missing data that may introduce bias and potentially compromise the accuracy of diagnosis and the interpretation of results. Second, the relatively small sample size, coupled with the absence of data from larger, multicenter cohorts, limits the generalizability of our findings and highlights the need for future studies with larger sample sizes and broader representation.

## 5 Conclusion

In summary, CT-PLB plays a pivotal role in the qualitative diagnosis of APH and PSP, serving as a safe and reliable diagnostic method. This technique enables the precise and minimally invasive acquisition of pathological specimens from pulmonary nodules, thereby facilitating accurate pathological diagnosis. By overcoming the limitations of imaging-based diagnostics, CT-PLB provides clinicians with robust evidence for diagnosis and significantly contributes to the development of personalized treatment strategies.

## Data Availability

The original contributions presented in the study are included in the article/supplementary material. Further inquiries can be directed to the corresponding authors.
